# The Effect of Vitamin D Supplementation on Insulin Resistance among Women with Polycystic Ovary Syndrome

**DOI:** 10.5935/1518-0557.20190032

**Published:** 2019

**Authors:** Saghar Salehpour, Sedighe Hosseini, Leila Nazari, Maryamsadat Hosseini, Nasrin Saharkhiz

**Affiliations:** 1 Preventative Gynecology Research Center (PGRC), Shahid Beheshti University of Medical Sciences, Tehran, Iran

**Keywords:** insulin resistance, vitamin D, polycystic ovary syndrome, fasting blood sugar, Iran

## Abstract

**Objective:**

To explore the effect of vitamin D supplementation on insulin resistance in a group of Iranian patients with polycystic ovary syndrome and vitamin D deficiency.

**Methods:**

This was a clinical trial conducted in a tertiary medical center in Tehran, the capital city of Iran, from May 2015 to September 2015. The participants included 41 women between 20 and 40 years of age with polycystic ovary syndrome based on the Rotterdam criteria and vitamin D deficiency. The fasting blood glucose and insulin levels, as well as serum 25-hydroxyvitamin D and homeostasis model assessment of insulin resistance (HOMA-IR) levels were measured at baseline and two months post treatment with a single dose of 300,000IU intramuscular vitamin D₃. The main outcome measures were plasma levels of vitamin D, fasting blood sugar and insulin levels, as well as insulin resistance.

**Results:**

The mean age of participants was 26.6±4.1. The serum level of 25-hydroxyvitamin D increased (5.7±1.77 to 16.34±8.99 ng/mL, *p*<0.001). The mean fasting blood glucose reading significantly decreased from 109.56±14.59mg/dL in pre-treatment to 103.71±13.72mg/dL post treatment (*p*=0.003). There was a significant decrease in the mean fasting serum insulin level from 8.52±5.48 mcU/mL before treatment with vitamin D to 7.07±5.03 (*p*=0.019) µU/mL after the treatment. The mean HOMA-IR, as a sign of insulin resistance, significantly decreased from 2.37±1.76 to 1.87±1.49, indicating less insulin resistance.

**Conclusions:**

A single injection of vitamin D significantly decreased serum insulin levels and insulin resistance among patients with polycystic ovary syndrome.

## INTRODUCTION

Polycystic ovary syndrome (PCOS), initially described in 1935 by Stein and Leventhal, is the most common endocrine disorder among females, affecting approximately 5%-20% of women in their reproductive age, depending on the criteria used (He *et al*., 2015; Asemi *et al*., 2015; Firouzabadi *et al*., 2012). In Iran, the prevalence of PCOS has been reported to be about 15% (Mehrabian *et al*., 2011). PCOS is a heterogeneous androgen excess disorder with different degrees of reproductive and metabolic dysfunctions (He *et al*., 2015), and it is characterized by hyperandrogenism, menstrual disorder and polycystic ovaries on ultrasound (Firouzabadi *et al*., 2012). Increased serum insulin levels, insulin resistance, glucose intolerance, and dyslipidemia are common among patients with PCOS (He *et al*., 2015).

A relatively higher prevalence of vitamin D deficiency (50% to 70%) is found among women with PCOS (Legro *et al*., 2004) compared to a prevalence of 20%-48% among the general adult population (He *et al*., 2015; Forrest & Stuhldreher, 2011; Hovsepian *et al*., 2011; Tangpricha *et al*., 2002). New studies have indicated a possible role for vitamin D in insulin secretion, as well as insulin resistance (He *et al*., 2015; Krul-Poel *et al*., 2013). Vitamin D deficiency might play a role in the pathogenesis of metabolic syndrome by increasing insulin synthesis and release, as well as insulin receptor expression (He *et al*., 2015; Ardabili *et al*., 2012). Insufficient vitamin D also disturbs extracellular and intracellular calcium balance, which may result in peripheral insulin resistance (Ardabili *et al*., 2012; Pittas *et al*., 2007; Jorde & Figenschau, 2009). Vitamin D deficiency is also followed by increased renin-angiotensin II expression, which might affect insulin resistance (Rammos *et al*., 2008).

Some previous studies have suggested that lower vitamin D levels might increase the risk of insulin resistance and metabolic disorder in women with PCOS, but their results are not conclusive (He *et al*., 2015; Verdoia *et al*., 2014; Wehr *et al*., 2009). Therefore, the objective of the present study was to evaluate the effects of vitamin D supplementation on insulin resistance among Iranian women with PCOS and vitamin D deficiency.

## MATERIALS AND METHODS

The present study was a non-controlled trial involving the pre and post treatment of women in the Taleghani Hospital Tehran, Iran from May 2015 to September 2015. Women between 20 and 40 years of age, with established PCOS based on the Rotterdam criteria, who also had vitamin D deficiency, entered the study. The ethics committee of the Shahid Beheshti University of Medical Sciences, Tehran, Iran, approved the study and all participants gave their written consent before entering the study. The present study complied with the tenets of the declaration of Helsinki. We also registered our study in the Iranian Registry of Clinical Trials with the registration number IRCT2014100619426N1. The exclusion criteria included Cushing's syndrome, adrenal hyperplasia, alcoholism, hyperprolactinemia, hypothyroidism, Diabetes Mellitus, androgen secreting tumors, pregnancy, lactation, and any systemic disorder, which can influence vitamin D levels, including malabsorption syndrome as well as liver and kidney diseases. Patients with a past of using drugs, which might affect the levels of vitamin D, such as calcium and vitamin D supplementation, antiepileptic drugs, oral hypoglycemic drugs and glucocorticoids, were excluded from the study.

A questionnaire which included height, weight, body mass index, history of infertility and menstrual status was filled for all participants before the start of the study and also 2 months post treatment with vitamin D. Fasting glucose, fasting insulin and 25-hydroxyvitamin D (25 (OH) D) levels were measured in all patients using a morning blood sample which was taken after a 12-hour overnight fast. We used a chemiluminescence immunoassay system to determine the serum insulin levels (Diasorin, Stillwater, MN, USA) and 25 (OH) D (DiaSorin SpA, Saluggia, Italy). The serum glucose levels were measured using a spectrophotometric method. All participants were then given a single dose of vitamin D (300,000 units) intramuscular and all tests were repeated again two months post treatment in the same manner. In addition, we calculated the glucose resistance index for all participants pretreatment and two months post-treatment. Insulin resistance was measured using the HOMA-IR method (Ardabili *et al*., 2012). The patients were advised to avoid taking vitamin D or calcium supplements during the study. The body mass index was calculated by weight in kilograms divided by the square of the height in meters (kg/m^2^).

### Statistical Methods

The normality of the response variables (vitamin D, fasting blood sugar, insulin, insulin resistance, insulin sensitivity index) was checked using the Kolmogorov Smirnov formula and the paired t-test was used to compare pre and post treatment readings if normality was established. For adjusting the effects of possible confounding parameters (age, Body Mass Index - BMI, initial vitamin D level) we repeated the measure analysis of variance (ANOVA) if data had normal distribution, and non-parametric tests, like the Wilcoxon test, were used if the data did not have normal distribution. All statistical tests were performed using the SPSS software version 24 (Armonk, NY: IBM Corp.). *p*-values below 0.05 were considered statistically significant.

Sample size calculation was performed using these parameters: given a type I error of 0.05 and power of 80% with effect size of 1.5 points change in the HOMA-IR based on the study by Ardabili *et al.* (2012) a sample size of 38 was calculated. We entered 41 patients to cover for probable patients lost on follow up.

## RESULTS

In total 41 patients with a mean age of 26.6±4.1 entered the study. All patients completed the study and no patient was lost on follow up. The minimum age was 20 and the maximum age was 36 years. The mean duration of infertility was 4.1±3.2 years and the mean weight and height were 67±12 kilograms and 161±5 cm, respectively (Table 1).

The mean BMI was 25.7±4.4. Other demographic findings of participants including their place of residence, menstruation and hirsutism findings are summarized in Table 2.

**Table 1:** Demographic findings of participants in the study

Parameter	Mean	Standard Deviation	Median	Minimum	Maximum
Age (Year)	26.6	4.1	26.0	20.0	36.0
Infertility duration (Year)	4.1	3.2	3.0	1.0	14.0
Weight (kg)	67	12	65	46	100
Height (cm)	161	5	160	150	175
BMI (kg/m^2^)	25.7	4.4	25.0	18.0	40.0

**Table 2:** Some other demographic findings of participants in the study

Parameter	Count	%
Residence (Tehran)	41	100.0%
Oligomenorrhea	25	61.0%
Regular Menstruation	16	39.0%
Hirsutism		
Absent	21	51.2%
Present	20	48.8%

The main findings of the study are summarized in Table 3. The vitamin D serum level significantly raised from a pretreatment mean of 5.07±1.77ng/mL to a post-treatment mean of 16.34±8.99ng/mL (*p*<0.001). The mean fasting blood glucose reading significantly decreased from 109.56±14.59mg/dL pretreatment to 103.71±13.72mg/dL post treatment (*p*=0.003). There was a significant decrease in the mean fasting serum insulin, from 8.52±5.48µU/mL pretreatment with vitamin D to 7.07±5.03 µU/mL post-treatment (*p*=0.019). The mean HOMA-IR, as a sign of insulin resistance, significantly decreased from 2.37±1.76 to 1.87±1.49, indicating lower insulin resistance (*p*=0.004).

**Table 3:** The changes in parameters related to glucose metabolism among PCOS patients two months after treatment with a single dose of vitamin D compared to pretreatment values

Parameter	Time	Mean	Standard Deviation	Median	Minimum	Maximum	*p*-value
Vitamin D ng/mL	Pre	16.34	8.99	13.27	3.04	39.97	<0.001
	Post	5.07	1.77	4.72	3.00	9.00	
Glucose mg/dL	Pre	109.56	14.59	109.00	78.00	136.00	0.003
	Post	103.71	13.72	102.00	77.00	137.00	
HOMA-IR	Pre	2.37	1.76	1.69	.54	9.40	0.004
	Post	1.87	1.49	1.49	.26	6.84	
Insulin mcU/mL	Pre	8.52	5.48	6.00	2.30	27.90	0.019
	Post	7.07	5.03	5.90	1.00	21.00	

## DISCUSSION

It has been indicated that among women with PCOS, those with vitamin D deficiency are more likely to have dysglycemia, when compared to those without vitamin D deficiency (He *et al*., 2105). Similarly, lower vitamin D levels have been related to abnormalities in glucose and insulin metabolism (Ardabili *et al*., 2012; Liu *et al*., 2009; Need *et al*., 2005), so we chose women with PCOS and low levels of vitamin D to see if a single dose supplementation with vitamin D can improve their glucose metabolism and insulin resistance.

There are several non-placebo controlled trials of vitamin D treatment on glucose metabolism among women with PCOS, with inconsistent results. In the present study, 1 intramuscular dose of cholecalciferol (300,000 IU) after 2 months caused significant reduction in insulin resistance, as per indicated by HOMA-IR among women with PCOS and vitamin D deficiency. Similar to our findings, a study by Selimoglu *et al*. on 11 insulin-resistant women with PCOS, reported that HOMA-IR significantly decreased three weeks after the administration of a single dose (300,000 IU) of cholecalciferol (Selimoglu *et al*., 2010). In our study the reduction in glucose and insulin levels were both statistically significant; however, Selimoglu *et al.*, reported a non-significant reduction of glucose and insulin levels among their patients (Selimoglu *et al*., 2010). In another study by Wehr *et al.*, fifty-seven PCOS women received 20,000 IU of cholecalciferol weekly for 24 weeks (Wehr *et al*., 2009). Anthropometric measures, oral glucose tolerance test, and blood analyses of endocrine parameters were performed at baseline and after 12 weeks and 24 weeks. They reported a significant decrease of fasting and stimulated glucose (*p*<0.05) and C-peptide levels (*p*<0.001) after vitamin D treatment (Wehr *et al*., 2009). Asemi *et al*. studied the effects of calcium plus vitamin D supplementation on glucose metabolism and lipid concentrations in overweight and obese vitamin D-deficient women with PCOS (Asemi *et al*., 2015). They found that co-supplementation, led to decreased serum insulin levels (*p*=0.03), homeostasis model of assessment of the insulin-resistance (HOMA-IR) score (*p*=0.04), and a significant rise in the quantitative insulin-sensitivity check index (QUICKI) (*p*=0.001) (Asemi *et al*., 2015).

In contrast in a study by Pal *et al*. (2012), twelve overweight and vitamin D deficient women with PCOS underwent a 2-hour oral glucose tolerance testing at baseline and following 3 month supplementation with vitamin D (daily dose of 3533 IU, increased to 8533 IU after the first 5 participants) and 530mg elemental Ca daily. They assessed plasma glucose, insulin, and insulin-resistance, and reported that the parameters of glucose homeostasis and insulin resistance remained unchanged (*p*>0.05). Also Kotsa *et al*. (2009) reported that treatment with alfacalcidol in patients with PCOS caused no statistically significant change in insulin sensitivity. In another study, Ardabili *et al*. evaluated the effects of vitamin D supplementation on insulin resistance in women with PCOS and vitamin D deficiency (Ardabili *et al*., 2012). In their study, the patients received 3 oral treatments consisting of 50,000 IU of vitamin D₃ (the case group) or a placebo (1 every 20 days) for 2 months. The fasting serum insulin and glucose levels and the insulin sensitivity and homeostasis model assessment of insulin resistance did not show a significant difference by the end of the study (Ardabili *et al*., 2012). Raja-Khan *et al*. (2014) studied the effects of high-dose vitamin D on insulin sensitivity in polycystic ovary syndrome (PCOS). They reported that insulin sensitivity was unchanged with high-dose vitamin D, but there was a trend toward decreased 2-hour insulin levels.

Our study had some limitations. We did not use the glucose clamp method (Ardabili *et al*., 2012), and we used the fasting level for evaluation of insulin sensitivity and secretion. Although we had a relatively small sample size, the significant results found in our study showed that the sample size seemed to be sufficient, and also we did not restrict our population to normal BMI, to avoid the confounding effect of weight loss after treatment, but we did not have significant weight loss in our study. Considering the controversial results of different studies in reducing the insulin resistance among PCOS women after supplementation with vitamin D, further experimentation with different doses of vitamin D and a longer intervention time are suggested in vitamin D-deficient women with PCOS to better support our findings.

In conclusion, it seems that a single injection of vitamin D significantly decreases serum insulin levels and insulin resistance among patients with polycystic ovary syndrome.
